# The Quanta Image Sensor: Every Photon Counts

**DOI:** 10.3390/s16081260

**Published:** 2016-08-10

**Authors:** Eric R. Fossum, Jiaju Ma, Saleh Masoodian, Leo Anzagira, Rachel Zizza

**Affiliations:** Thayer School of Engineering at Dartmouth, Dartmouth College, Hanover, NH 03755, USA; Jiaju.Ma.TH@dartmouth.edu (J.M.); Saleh.Masoodian.TH@dartmouth.edu (S.M.); Leo.Anzagira.TH@dartmouth.edu (L.A.); rzizza111@gmail.com (R.Z.)

**Keywords:** photon counting, image sensor, quanta image sensor, QIS, low read noise, low power

## Abstract

The Quanta Image Sensor (QIS) was conceived when contemplating shrinking pixel sizes and storage capacities, and the steady increase in digital processing power. In the single-bit QIS, the output of each field is a binary bit plane, where each bit represents the presence or absence of at least one photoelectron in a photodetector. A series of bit planes is generated through high-speed readout, and a kernel or “cubicle” of bits (x, y, t) is used to create a single output image pixel. The size of the cubicle can be adjusted post-acquisition to optimize image quality. The specialized sub-diffraction-limit photodetectors in the QIS are referred to as “jots” and a QIS may have a gigajot or more, read out at 1000 fps, for a data rate exceeding 1 Tb/s. Basically, we are trying to count photons as they arrive at the sensor. This paper reviews the QIS concept and its imaging characteristics. Recent progress towards realizing the QIS for commercial and scientific purposes is discussed. This includes implementation of a pump-gate jot device in a 65 nm CIS BSI process yielding read noise as low as 0.22 e− r.m.s. and conversion gain as high as 420 µV/e−, power efficient readout electronics, currently as low as 0.4 pJ/b in the same process, creating high dynamic range images from jot data, and understanding the imaging characteristics of single-bit and multi-bit QIS devices. The QIS represents a possible major paradigm shift in image capture.

## 1. Introduction

The Quanta Image Sensor (QIS) was conceived in 2004 and published in 2005 [1,2,3,4] as a forward look at where image sensors may go in the 10 to 15-year future as progress in semiconductor device technology would allow sub-diffraction limit (SDL) pixels to be readily implemented, and advancement in circuit design and scaling would permit greater pixel throughput at reasonable power dissipation levels. Active research began in 2008 at Samsung (Yongin, Korea) [5] but was short lived due to economic pressure in that period. Research began anew at Dartmouth in 2011 and was supported from 2012 to the present by Rambus Inc. (Sunnyvale, CA, USA). Since 2011, progress has been made in pixels, readout circuits, and image formation [6,7,8,9,10,11,12,13,14,15,16,17,18,19,20,21,22,23,24,25,26]. In this paper, the QIS concept and progress to date is reviewed.

In the QIS, SDL pixels (e.g., 200 nm–1000 nm pitch) are sensitive to single photoelectrons, so that the presence or absence of one electron will result in a logical binary output of 0 or 1 upon readout. The specialized pixel, called a “jot”, (Greek for “smallest thing”) needs only the smallest full-well capacity (FWC). It is envisioned that a QIS will consist of hundreds of millions or perhaps billions of jots read out at perhaps 1000 fields per second, resulting in a series of bit planes, each corresponding to one field. The bit data can be thought of as a jot data cube, with two spatial dimensions (x and y) with the third dimension being time.

Output image pixels are created by locally processing the jot data cube to create a representation of local light intensity received by the QIS. Since this processing occurs post-capture, great flexibility is afforded in choosing the effective spatial dimensions of a pixel as well as its temporal dimension (e.g., digital integration time). Conceptually, if the bit data is an accurate representation of the collection and counting of photoelectrons, the combining of jot data is noiseless, allowing functionality such as time-delay-and-integration (TDI) to be performed post-capture on the data in an arbitrary track direction. In fact, different tracks can be used in different portions of the image. Indeed, even relative motion of objects within the field of view can be determined and refined iteratively to optimize the image generation process. Spatial and temporal resolution can also be adjusted for different portions of the image.

After the QIS concept was introduced in 2005, the concept was applied for use with single-photon avalanche detectors (SPADs) by the group at the University of Edinburgh [27,28,29,30] as published starting in 2014 as part of their SPAD research program. Other work on SPADs published in 2005 shows concurrent conception of some of the same ideas [31], and in 2009 a “gigavision camera” using binary pixels was proposed by researchers from École Polytechnique Fédérale de Lausanne (EPFL) [32,33,34,35,36]. Some characteristics of the QIS can be traced to photographic film as reported in 1890 [37] and other characteristics were observed in photon-counting devices implemented using vacuum tubes and solid-state devices [38,39,40,41,42,43]. Progress in other devices achieving sub-electron and deep sub-electron read noise has mainly been made in the past few years [44,45,46,47,48,49,50,51].

Possible applications of the QIS include scientific low-light imaging such as in life sciences (e.g., microscopy), defense and aerospace, professional and consumer photography and cinematography, multi-aperture imaging, cryptography (quantum random number generation), direct detection of low-energy charged particles, and others.

To realize the QIS in a convenient form, several theoretical and technological issues require exploration. These are: (1) image formation algorithms that yield high quality images; (2) understanding the imaging characteristics of QIS devices; (3) the implementation of pixels (jots) that enable photon counting; (4) low-power readout of high volumes of data (readout of a 1 Gjot sensor at 1000 fps yields a data throughput rate of 1 Tb/s) and (5) on-focal-plane processing to reduce the data volume. Exploration of these issues of the course of the last several years has led to significant advancement in the first four areas with the fifth just being explored now. This paper reviews this progress. Additional details may be found in the cited references.

## 2. Creating Images from Jots

Readout of jots results in a bit cube of data, with two dimensions representing spatial dimensions of the field-of-view of the sensor, and the third representing time. Each bit-plane slice is a single readout field. For single-bit QIS devices, the jot data cube is binary in nature. For multi-bit QIS, the jot data cube consists of words of bit length corresponding the readout quantization bit depth described in more detail below.

**Figure 1:**
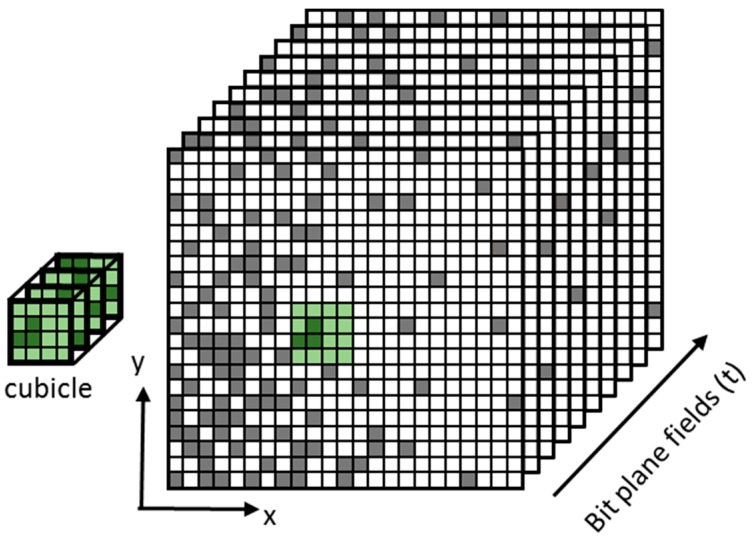
Conceptual illustration of a jot data cube and a 4 × 4 × 4 cubicle subset.

**Figure 2:**
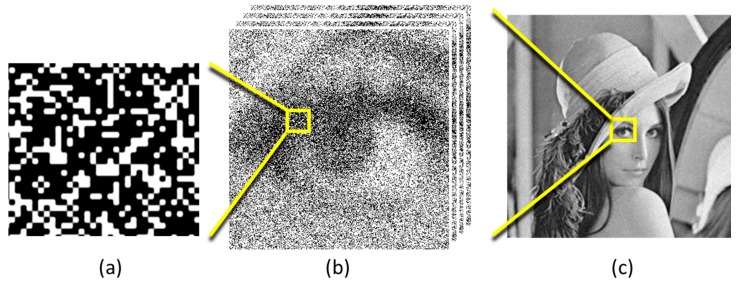
Simulation of (**a**) raw jot data; (**b**) same at lower magnification; (**c**) after processing cubicles to form grey scale image. From [16].

In perhaps the simplest form, image pixels can be created from the sum of a small x-y-t “cubicle” of bits from the jot data cube (see Figure 1). The dimensions of the cubicle determine the spatial and temporal resolution of the output image, and all jot values within the cubicle are weighted equally. The maximum signal-to-noise ratio (SNR) of the image pixel (assuming an ensemble of pixels created from the same illumination and readout conditions) is determined by the size of the cubicle. For example, a cubicle of size 16 × 16 × 16 of single-bit QIS jots summed together would have a maximum value of 4096 and a maximum SNR of $\sqrt{4096}=64$. Illustration of image formation from simulated jot data is shown in Figure 2. Note that cubicles do not have to have equal dimensions in x, y and t. Furthermore, all image pixels need not be formed from the same sized cubicles, and their cubicles may overlap. These choices can be made post-capture and on an output frame-by-frame basis to optimize particular imaging aspects such as the trade-off between image SNR and spatial-temporal resolution. An example of this processing was recently demonstrated using a QIS-like device with SPAD-based jots [28] where the pixels near the edges of rotating fan blade were processed differently than either the slowly varying body of the blades or the static background.

Using cubicles that have a non-orthogonal trajectory in the time dimension can be used for performing operations analogous to time-delay and integration (TDI) but along an arbitrary track direction [6]. Different objects in the scene with different motion trajectories could be processed with independent tracks to improve SNR. The utility of such processing is enhanced by deep sub-electron read noise in the QIS and quantizer, so that the noise is inherently low and does not accumulate like $\sqrt{M}$, where *M* is the ensemble size of jots summed together.

Measuring photon flux with a single-pixel photon-counting photomultiplier tube was reported as early as 1968 [38]. The generation of an image with improved SNR from a series of readout fields goes back to 1985 when photon-counting detectors were used this way for astronomy purposes [39,40]. The technique was also applied to CCDs with built-in avalanche multiplication [43]. Similar operations with CMOS image sensors (CIS) were envisioned as early as 1998 [52] and proposed as a digital integration sensor (DIS) [7]. The technique was also applied in SPAD arrays [31]. SPAD arrays operating as QIS devices were demonstrated in [30]. Following the introduction of the early QIS concept, the “gigavision” binary image sensor was proposed in 2009 [33,34]. Image formation from bits in the binary sensor was explored mathematically by EPFL [36] and by Harvard [53]. These investigations concerned a cubicle that was essentially 1 × 1 × *N*, that is, one pixel read out *N* times.

QIS devices use both spatial and temporal sampling to create one image pixel using sub-diffraction limit pixel sizes [2], although binning multiple pixels to improve SNR at the expense of spatial resolution dates back to at least the early days of CCDs in the 1970’s. Uniform weighting and other weight distributions applied to jots of the bit cube to form image pixels have been explored by Zizza [16]. It was found that there was little apparent impact on image quality between non-uniformly and uniformly weighted cubicles, and modulation transfer function (MTF) and SNR were also not significantly impacted. It was also found that the EPFL and Harvard algorithms for creating images from jots worked well for single, static images, but when processing time and latency are considered for continuous image acquisition, simple summation of cubicle data is preferred.

## 3. Imaging Characteristics

### 3.1. Hurter-Driffield Characteristic Response (D-LogH)

In the QIS, the statistical nature of the arrival of photons and the photoelectrons they produce are well described by Poisson arrival statistics. For convenience we define the quanta exposure *H* as the average number of photoelectrons collected by a jot over an integration period, which depends on factors such as the incident photon flux, effective jot area, quantum efficiency, carrier collection efficiency and integration time. The probability of there being *k* photoelectrons is given by the Poisson mass function:

(1) $$P[k]=\frac{e^{-H}H^{k}}{k!}$$

The probability that there are no photoelectrons is $P[0]=e^{-H}$, and the probability of at least one photoelectron is $P\left[k>0\right]=1-P\left[0\right]=1-e^{-H}$. In the single-bit QIS, reading out no photoelectrons is a logic “0” and reading out one or more photoelectrons is set as a logic “1”. Essentially, the full-well capacity (FWC) of the single-bit QIS is 1 e−. The bit density *D* of jots (fraction per bit ensemble that have logic value “1”) gives rise to an S-shaped curve if *D* is plotted as a function of log(*H)*, reflecting the relationship [3,9]:

(2a) $$D=1-e^{-H}$$

**Figure 3:**
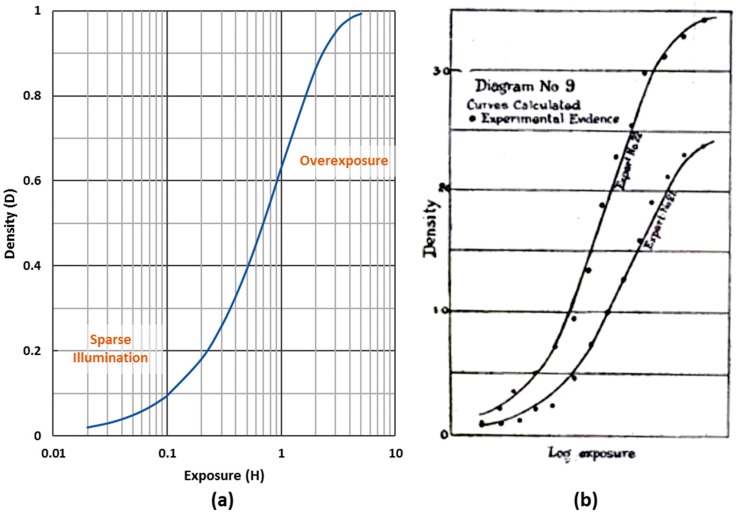
(**a**) Single-bit QIS bit density as a function of quanta exposure, in *D*-log(*H*) format. Sparse and over exposure regions are labelled [9]; (**b**) *D*-log(*H*) curve for photographic plates as reported by Hurter and Driffield in 1890 [37].

**Figure 4:**
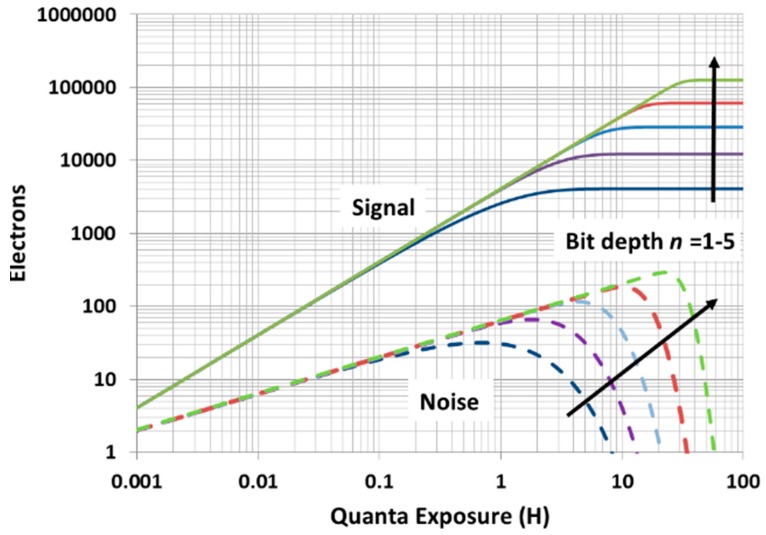
Log signal and noise as a function of log exposure for multi-bit QIS jots with varying bit depth. The signal is the expected sum over 4096 jots (e.g., 16 × 16 × 16). Saturation signal is 4096 (2*^n^* − 1) (From [14]).

This characteristic QIS-response curve is shown in Figure 3a. This behavior is similar to the behavior of an ensemble of avalanche detectors in a Si photomultiplier and also observed in SPADs and in subsequent analysis of binary sensors [33,34,41]. The standard deviation of *D* (or noise, *σ*) is given by [9]:

(2b) $$\sigma =\sqrt{e^{-H\;}\left[1-e^{-H}\right]}$$

as shown in Figure 4.

The statistical nature of photoelectron counting (or essentially photon counting if the efficiency factors above are close to unity) is the same as that which gives rise to the *D-*log(*H)* nature of film exposure reported by Hurter and Driffield in 1890 [37] due to the statistical exposure of film grains, as shown in Figure 3b. *Henceforth, the asymptotic response for Figure 3a,b is referred to as the Hurter-Driffield response curve in their honor, whether referring to film, SPADs or QIS jots*. In the case of Ag-I film grains, about 3 photoelectrons are required to result in exposure leading to slightly different slopes [9,33,42]. In fact, many photographers and cinematographers desire this non-linear behavior that results in a response curve with good overexposure latitude [54]. The curve shape is determined by the underlying statistical physics of exposure and threshold for creating jots with value “1”, and is not influenced strongly by circuit or device performance. Further, the exposure-referred signal-to-noise ratio (*SNR_H_*) as a function of exposure is well-behaved [9], unlike that which is found in conventional image sensors operating in high dynamic range mode, and which suffer from one or more large dips in *SNR_H_* as the exposure is increased (e.g., [7]).

### 3.2. Flux Capacity

An important figure of merit for QIS devices is *flux capacity*. Flux capacity $\phi_{w}$ is defined as the nominal maximum photon flux that results in *H* = 1. It is dependent on the density of jots *j* in the image sensor (jots/cm^2^), the readout field rate $f_{r}$, the shutter duty cycle δ and the effective quantum efficiency $\bar{\gamma }$ according to [14]:

(3) $$\phi_{w}=jf_{r}/\delta \bar{\gamma }$$

For photography and cinematography, high flux capacity is required so that the QIS does not saturate under normal imaging conditions. Note that the QIS can handle exposures for *H* > 1 without saturating, typically 5× higher due to its overexposure latitude, but *H* = 1 is taken for convenience. For example, with a jot pitch of 500 nm, 1000 fps field rate, unity duty cycle and 50% avg. QE, the flux capacity is 8 × 10^11^ photons/cm^2^/s which at F/2.8, QE = 50%, lens T = 80%, scene R = 20%, corresponds to ~400 lux at the scene (yielding *H* = 1 at the sensor). It can be seen that high jot density and high field readout rate are driven by flux capacity and not necessarily by improved spatial nor temporal resolution of the final image, although these are additional benefits. The sub-diffraction jot pitch requires use of advanced-node processes that are expensive and difficult to access today. The high readout rate creates challenges in controlling power dissipation in the readout circuit.

### 3.3. Multi-Bit QIS

To further improve flux capacity, the multi-bit QIS was proposed. In the multi-bit QIS, the readout result can result in 2*^n^* states, where *n* is the readout bit depth (a single-bit QIS is, in essence, a special case of a multi-bit QIS with *n* = 1). For example, if *n* = 2 then 4 possible states can be considered, (1) no photoelectron, $P[0]=e^{-H}$ as before, now coded logically as “00”; (2) one photoelectron, $P[1]=He^{-H}$ now coded logically as “01”; (3) two photoelectrons, $P[2]=H^{2}e^{-H}/2$ coded logically as “10”; and (4) 3 or more photoelectrons, $P\left[k>2\right]=1-e^{-H}-He^{-H}-H^{2}e^{-H}/2$, coded logically as “11”. During readout of the signal from the jot, each of these states must be discriminated, such as by using a 2b analog-to-digital converter (ADC). The multi-bit QIS has a *FWC* given by $2^{n}-1$ and the flux capacity $\phi_{wn}$ is increased to:

(4) $$\phi_{wn}=jf_{r}\left(2^{n}-1\right)/\delta \bar{\gamma }$$

The Hurter-Driffield response is modified by multi-bit QIS readout resulting in higher saturation signal, less non-linearity and less overexposure latitude [9,14]. The expected number of electrons read out from a multi-bit QIS jot is given by <*k*> where:

(5) $$<k>=\overset{FWC}{\underset{k\;=\;0}{\sum }}k\cdot \;P\left[k\right]+\overset{\infty }{\underset{k\;=\;FWC\;+\;1}{\sum }}FWC\cdot \;P\left[k\right]\;=\;FWC\left[1-\overset{FWC}{\underset{k\;=\;0}{\sum }}\left(1-\frac{k}{FWC}\right)\cdot \;P\left[k\right]\right]$$

The variance in the number of electrons is:

(6) $$\sigma^{2}=\;<k^{2}>-\;<k{>}^{2}$$

where $<k^{2}>$ is given by:

(7) $$<k^{2}>\;=\overset{FWC}{\underset{k\;=\;0}{\sum }}k^{2}\cdot \;P\left[k\right]+\overset{\infty }{\underset{k\;=\;FWC\;+\;1}{\sum }}FWC^{2}\cdot \;P\left[k\right]\;=\;FWC^{2}\left[1-\overset{FWC}{\underset{k\;=\;0}{\sum }}\left(1-\frac{k^{2}}{FWC^{2}}\right)\cdot \;P\left[k\right]\right]$$

The multi-bit signal summed over an ensemble of *M* jots is M<k>, and the noise (standard deviation) is $\sqrt{M\sigma^{2}}$.

Consider an ensemble of 4096 jots. For a single-bit QIS, the maximum signal obtained by adding together the logical readout signal from each jot (0 or 1) is 4096. For the 2-bit QIS, the maximum signal is increased by three-fold ($2^{n}-1)$ to 12,288 and the noise characteristic rolls off more steeply. The summed signal of an ensemble of 4096 multi-bit jots is shown in Figure 4. The predicted signal and noise vs. exposure relationship for a single-bit QIS was first experimentally verified by Dutton et al. [29].

### 3.4. Signal-to-Noise Ratio (SNR) and Dynamic Range (DR)

The use of exposure-referred *SNR* is useful for non-linear devices, especially when intrinsic signal noise drops near saturation. The *SNR_H_* for normal readout of a single-bit QIS is given by [9]:

(8) $$SNR_{H}=\sqrt{M}\frac{H}{\sqrt{e^{H}-1}}$$

where *M* is the number of jots in the ensemble or cubicle used for the read signal sum. This assumes the read noise is low enough that the readout bit error rate (BER) [9,14] does not significantly affect the sum—a reasonable assumption for the QIS target read noise of less than 0.15 e− r.m.s. [9,21]. For normal readout, the *SNR_H_* reaches a maximum value at H≅1.6 and the maximum value of *SNR_H_* is approximately $0.8\sqrt{M}$. For multi-bit QIS, maximum *SNR_H_* increases as ~$\sqrt{FWC}$.

**Figure 5:**
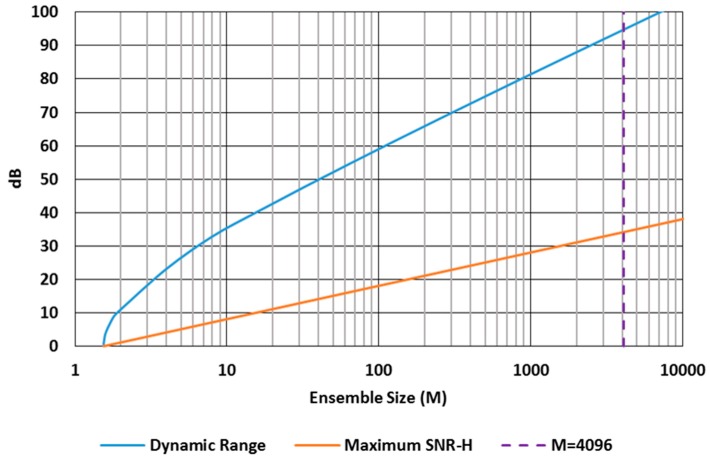
Single-bit QIS normal readout dynamic range (blue) and maximum *SNR_H_* as a function of ensemble or cubicle size *M*. For convenience, *M* = 4096 is highlighted by the purple dashed line. Note that *M* is confined to integer values despite the continuous nature of the curves in this figure.

Dynamic range (*DR*) for the QIS is defined as the range between low signal *H_min_* where *SNR_H_* = 1 (essentially where the ensemble of read out jots has a total sum of one photoelectron) and high signal *H_max_* where *SNR_H_* drops back down to unity and lower due to saturation [9]. The *DR* depends on the size of the ensemble—more jots, higher dynamic range, and the *DR* scales approximately as *M*, where *M* is the number of jots in the ensemble. The *DR* and the maximum value of *SNR_H_* are shown in Figure 5 as a function of ensemble size, calculated using the expressions derived in [9]. At *M* = 4096, for example, the *DR* is 95 dB and the maximum value of *SNR_H_* is 34 dB. This can be compared to a conventional CIS with FWC of 4096 e− and read noise of 1.5 e− r.m.s. The CIS would have a *DR* of 68 dB and maximum SNR of 36 dB assuming linear response. Increasing the bit depth does not substantially increase the *DR* due to the reduction in non-linearity (overexposure latitude) as the bit depth is increased. For example, going from single-bit to 2b QIS (3× increase in FWC) only increases the *DR* by approximately 3 dB.

The response of the single-bit QIS is only linear for H≲0.1 with increasing non-linearity above this exposure level. The non-linearity is a desirable feature for photography and cinematography, but not as much for some other applications, and undesirable for most scientific photon-counting applications. In the latter case, the linear response can be retrieved from the non-linear signal or the exposure must be kept so H≲0.1. Multi-bit QIS has a larger range of linearity as can be readily seen in Figure 4. As was noted in [14] multi-bit signals can be easily transformed into lower bit depth signals, or single-bit signals, by post-readout digital signal processing. This permits some flexibility in the response curve by trading FWC or flux capacity for linearity.

### 3.5. High Dynamic Range (HDR)

**Figure 6:**
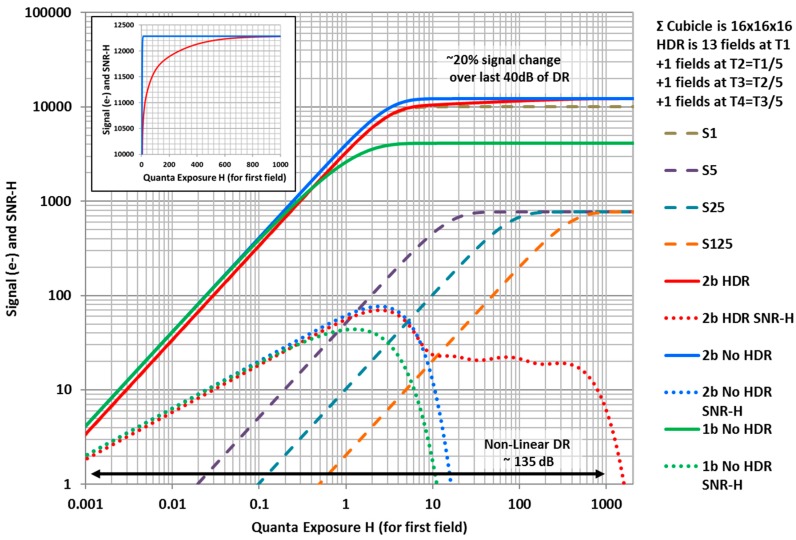
Log signal vs. log exposure for a 2b QIS operated in normal readout mode (blue) and high dynamic range mode (red), showing extension of the dynamic range by approximately 40 dB. Inset shows linear-scale response curves for the extended exposure range.

Since the QIS consists of multiple fields of jot data that may be combined in a cubicle ensemble, it is possible to have a different electronic shutter duty cycle or “speed” for each field or time slice. Thus some time slices can have high flux capacities allowing capture of brighter portions of scenes without saturation. A similar idea has been used in CMOS image sensors for many years [55] for high dynamic range (HDR) imaging, although it can suffer from imaging artifacts due to relative motion of the scene between captured fields. The higher field readout rate of the QIS will ameliorate some of those artifacts. The Hurter-Driffield response characteristics of the QIS help reduce SNR “dips” caused by the fusion of multiple fields of data taken with different shutter speeds [9]. Multi-bit QIS devices can also be operated in HDR mode. For example, consider 2b-QIS output formed from a 16 × 16 × 16 cubicle. In normal readout mode summing all 16 fields, each with 100% shutter duty cycle, the *DR* is approximately 98 dB. In an HDR mode, with summing a cubicle where 13 fields are exposed with 100% shutter duty cycle, 1 field at 20% duty cycle, 1 field at 4% duty cycle, and 1 field at 0.8% duty cycle, the dynamic is extended to approximately 135 dB as illustrated in Figure 6. Figure 6 shows the log signal vs. log exposure characteristic of a 1b QIS, 2b QIS and their attendant *SNR_H_* when the 16 fields in the cubicle all have 100% duty cycle and are summed (green and blue respectively). For the 2b QIS, the maximum signal is 3 e− per jot leading to a maximum sum of the cubicle of 3 × 4096 = 12,288. Also shown in the figure are the 2b QIS cubicle sums vs. exposure for the 4 component fields. S1 shows the sum of 13 fields taken with 100% shutter duty cycle. The number of fields should be large in order to capture low light detail in the image with good SNR. The three following fields’ cubicle sums S5, S25 and S125 are taken with 20%, 4%, and 0.08% duty cycles respectively—essentially 1/5, 1/25, and 1/125 relative shutter speeds. The sum of all these fields of the cubicle is the 2b HDR signal vs. exposure characteristic (red) along with its attendant *SNR_H_*. The latter has one large *SNR_H_* dip from its peak of ~37 dB to a plateau of 27 dB in the extended range. While on a log scale, the HDR curve (red) looks similar to the normal readout curve (blue), the inset shows a linear-linear plot of the extended range, showing significant contrast for the HDR response. Different transfer curves can be generated by varying the relative duty cycles and number of fields and this particular set of signal vs. exposure curves are just one example set.

## 4. Read Noise and Counting Error Rates

### 4.1. Read Noise and Readout Signal Probability

Counting photon or photoelectrons requires deep sub-electron read noise (DSERN), that is, read noise less than 0.50 e− r.m.s. It has been suggested that 0.30 e− r.m.s. read noise is sufficient for many photon-counting applications [47,56,57] however accurate counting with low error rate under low exposures (e.g., *H* < 0.2) requires read noise less than 0.15 e− r.m.s. [21].

When both read noise and conversion gain variation is considered for an ensemble of jots that are read out, the probability distribution of readout voltages is given by:

(9) $$P\left[U\right]=\overset{\infty }{\underset{k=0}{\sum }}\frac{P[k]}{\sqrt{2\pi \sigma_{k}^{2}}}exp\left[-\frac{{\left(U-k\right)}^{2}}{2\sigma_{k}^{2}}\right]$$

where *U* is the readout signal normalized by mean conversion gain (in electron number), *u_n_* is the read noise (in e− r.m.s.), and where $\sigma_{k}$ is given by:

(10) $$\sigma_{k}≜\sqrt{u_{n}^{2}+{\left(k\sigma_{CG}/\bar{CG}\right)}^{2}}$$

and $\sigma_{CG}$ is the standard deviation of conversion gain in the ensemble, and $\bar{CG}$ is the mean conversion gain in the ensemble.

**Figure 7:**
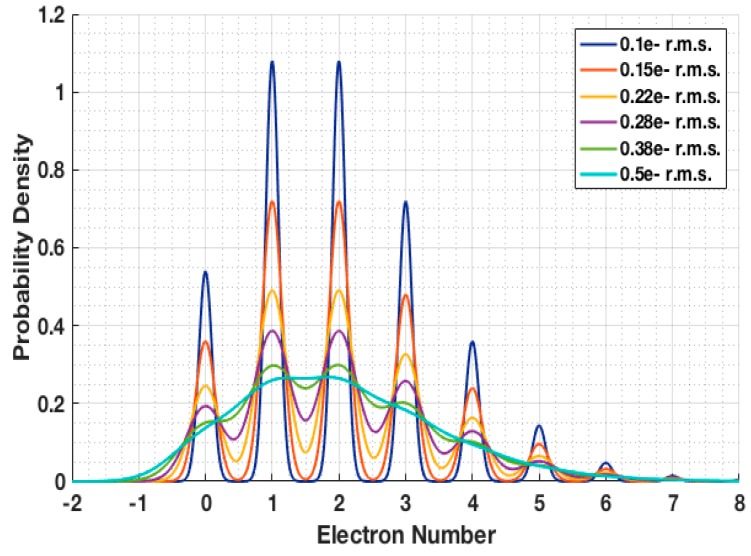
Readout signal probability density as a function readout signal for a quanta exposure *H* = 2 and for various read noise levels. Examples with CG variation are shown in [21,22].

An example of the distributions arising from different levels of read noise is shown in Figure 7 Electron number quantization is seen for read noise in the deep sub-electron range. Using a fine resolution ADC, plots of frequency of occurrence vs. readout voltage can be made for experimental jot devices under illumination and are called photon-counting histograms (PCH). The ratio of valley amplitude to peak amplitude, called valley-peak modulation (VPM) can be used to experimentally determine read noise from the PCH, the peak spacing can be used to determine conversion gain, and the relative peak heights can be used to determine exposure [22].

### 4.2. Quantization and Bin Counts

In a single-bit QIS, the threshold level *U_T1_* for setting the output to a logic “1” is *U_T1_* = 0.5, so that the digital output is “1” for *U* > 0.5 and otherwise “0”. For multi-bit QIS, additional thresholds are set at integer increments above *U_T1_* (e.g., *U_T2_* = 1.5, U*_T3_* = 2.5, etc.). All read out signals lying between two adjacent thresholds (a bin) result in the count for that bin $C_{N}$ being incremented by one. False positive counts are generated when a read out signal is misquantized into the wrong bin due to noise and conversion gain variation. These false positives (and their corresponding false negatives) give rise to an error in the total count.

An ensemble of *M* jots results in a total of *M* counts spread across the *2^n^* bins of a multi-bit QIS. The total count in each bin can be used to determine the expected total number of photoelectrons collected by the ensemble $N_{TOT}$ such that [21]:

(11) $$N_{TOT}=M\overset{\infty }{\underset{N\;=\;0}{\sum }}N\cdot C_{N}$$

For $\sigma_{k}≲$ 0.50 e− r.m.s., the expected count in each bin for a single jot is given by:

(12) $$C_{N}≅\overset{N\;+\;1}{\underset{k\;=\;N\;-\;1}{\sum }}\frac{1}{2}P[k]\left[\mathrm{erf}\left(\frac{N+\frac{1}{2}-k}{\sigma_{k}\sqrt{2}}\right)-\mathrm{erf}\left(\frac{N-\frac{1}{2}-k}{\sigma_{k}\sqrt{2}}\right)\right]$$

except for bin 0 and last bin $2^{n}-1$ where in the former the bin extends to U=−∞, and in the latter to U=+∞, so that:

(13) $$C_{0}=\frac{1}{2}\overset{\infty }{\underset{k\;=\;0}{\sum }}P[k]\left[\mathrm{erf}\left(\frac{\frac{1}{2}-k}{\sigma_{k}\sqrt{2}}\right)+1\right]$$

and:

(14) $$C_{2^{n\;}-\;1\;}=\frac{1}{2}\overset{\infty }{\underset{k\;=\;0}{\sum }}P[k]\left[1-\mathrm{erf}\left(\frac{(2^{n}-1)-\frac{1}{2}-k}{\sigma_{k}\sqrt{2}}\right)\right]$$

In [21] it was found that for higher quanta exposures (*H* > 0.2), the count was not significantly affected by (deep sub-electron) read noise nor conversion gain variation in the ensemble, assuming *M* was sufficiently large, and the non-linear Hurter-Driffield response dominated counting error in a predictable way. For lower quanta exposure, systematic count error was introduced by read noise. Essentially under sparse illumination conditions (*H* < 0.1), even a small amount of read noise can cause excess counting by the occasional misquantization of the dominant “0” signal as “1”. This systematic count error can result in counting rate error of 34% for *H* = 0.1 and read noise of 0.30 e− r.m.s. yet nearly no error at a read noise of 0.20 e− r.m.s. The systematic counting rate error increases dramatically for lower exposures, strongly indicating that for applications requiring accurate photon counting in this realm, read noise should be 0.15 e− r.m.s. or smaller.

**Figure 8:**
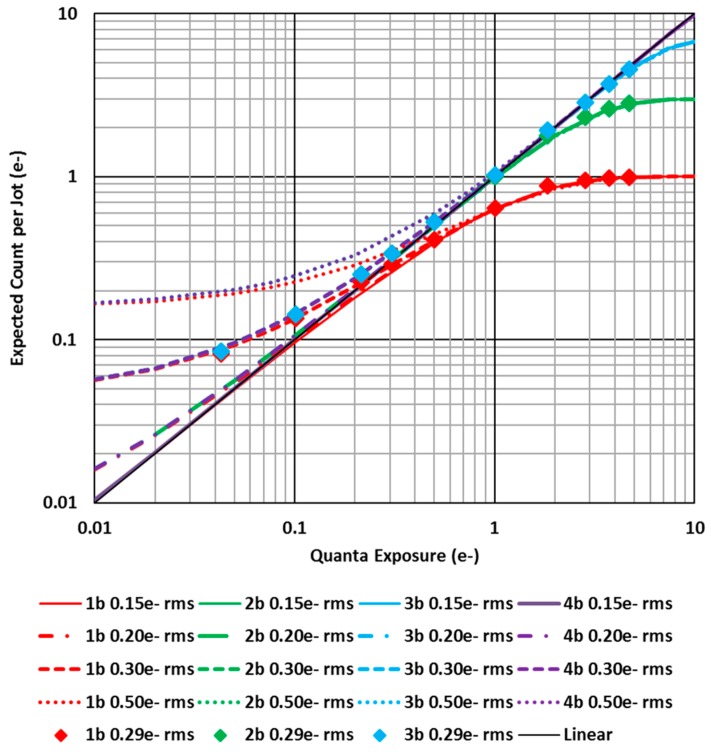
Expected count vs. quanta exposure for various bit depths and read noise levels. Experimental data is shown by the diamond symbols (after [21]).

Count vs. quanta exposure is shown in Figure 8. Ideally the count should be equal to the quanta exposure leading to a linear relationship shown by the diagonal gray line (mostly obscured). For a 4b QIS with read noise of 0.15 e− r.m.s., (purple solid line), the count is nearly indistinguishable from the linear relationship. However, for higher read noise levels, significant systematic departure from the ideal behavior under sparse illumination conditions can be observed, independent of bit depth. At higher exposures, the Hurter-Driffield response dominates the non-linear behavior independently of read noise. In all cases, the impact of conversion gain variation in the ensemble is negligible if *M* is sufficiently large, and if not, then photoresponse non-uniformity can be an issue as in conventional CIS devices.

The expected count in Equations (11)–(14) can be used to estimate the count for an ensemble of *M* jots. For example, consider a single-bit QIS array of jots with pitch of 1 µm. An ensemble of 100 jots, formed from 10 × 10 × 1 cubicle would cover an area of size 10 µm × 10 µm. For a quanta exposure *H* = 0.01, the ideal expected count from the ensemble would by $N_{TOT}=1$. Using Equations (11)–(14) or Figure 8 with *H* = 0.01, and read noise of 0.15 e− r.m.s., one obtains an expected count of 100 × 0.0104 = 1.04 e−, but for a higher read noise of 0.30 e− r.m.s., one obtains an expected count of 100 × 0.0568 = 5.68 e−. It is noted that this systematic error is different in nature than a typical manifestation of read noise which leads to a correct average value over a large number of samples but with some standard deviation or noise. In this case, the average readout value itself is offset.

Sub-electron (voltage) quantizer resolution (e.g., 0.05 e−) may be used to provide more accurate counting in the presence of higher read noise, by computing the mean signal of a larger number of samples and converting to electrons, as is done conventionally and which was used to calibrate the horizontal axis of Figure 8. However this requires a more accurate ADC, higher power, and likely slower field readout rate.

## 5. Jot Device

### 5.1. Background and Motivation

The ultimate goals of a jot device include small pitch size (200 nm–500 nm), low read noise (<0.15 e− r.m.s.), low dark current (<1 e−/s), small FWC (1–100 e−) and strong compatibility with a CIS fabrication line. One big difference between a jot and a conventional CIS pixel is its deep sub-electron read noise and photoelectron counting capability. A conventional CIS often has voltage read noise higher than 100 μV r.m.s. and CG lower than 100 μV/e−, yielding read noise higher than 1 e− r.m.s. Higher CG and lower voltage noise reduce input-referred read noise. As a possible candidate for a jot device, SPADs are widely used for photon counting [see this Special Issue]. Through the avalanche multiplication effect, it can provide a higher CG (>1 mV/photoelectron) and low read noise (<0.15 e− r.m.s.). It has been used to demonstrate the QIS concept and showed interesting results [27,28,29,30]. Unfortunately, its relatively large size (typically 5–10 μm pitch) limits flux capacity and resolution [32], high electric fields result in high dark count rate (~1000 counts/s/pix), low fill factor and dead time reduce photon detection efficiency, and manufacturing yield is lower than conventional CIS process on a pixel-by-pixel basis. Electron-multiplying CCD (EMCCD) technology provides high CG by an electron multiplication process and is able to achieve 0.45 e− r.m.s. average read noise [49]. But similar to SPAD arrays, it has a high dark current due to thermal generation of carriers under high electric fields. EMCCDs also have relatively low frame rates as the signal is read out by CCD circuitry. We have considered these devices and other devices such as floating-base bipolar transistors, as candidates [11].

In consideration of fabrication feasibility, we started the jot design based on a conventional intra-pixel charge transfer approach similar to conventional CIS 4T pixels with a “pinned photodiode” due to its mature fabrication process, low dark current and high quantum efficiency, which also provides the jot device with good compatibility to many techniques developed in CIS, such as BSI, shared readout and stacked process. For example, in a BSI device, fill factor is very high, nearly unity, and backside treatments to reduce reflection losses are well known from the CCD era. Carrier collection efficiency can also high, depending on detailed device design and the funneling of carriers to the storage well. The readout introduces minimal dead time compared to SPADs, and may be as low as 0.004% in a gigajot sensor. A typical CIS readout chain includes an in-pixel source follower (SF), a correlated double sampling (CDS) circuitry, a high analog-gain amplifier and an ADC. Voltage noise is added to the voltage signal by each readout component before the signal is digitized in the ADC, and in standard practice, it is best to add gain earlier in the signal chain to ameliorate the impact of downstream noise components. Generally, in a low-noise (1 e− r.m.s. to 1.5 e− r.m.s.) CIS, the in-pixel SF contributes most of the input-referred voltage noise, typically 100–200 μV r.m.s. An in-pixel common source amplifier can provide a higher than unity gain and suppress latter noise sources without increasing the pixel size, but it also generates high gain variation [45], which can be detrimental for multi-bit QIS application. The major noise components in an in-pixel SF are 1/*f* noise and random-telegraph signal (RTS), and both appear to be related to the carrier capture and emission process of surface interface traps, either at the gate oxide-semiconductor interface, or due to shallow trench isolation sidewalls [58], although other sources of 1/*f* noise such as turbulent flow have been suggested [59,60]. Buried-channel SF and correlated multiple sampling (CMS) techniques [61] were applied to reduce the SF noise, and 35 μV r.m.s. voltage noise was achieved, but with a relatively low CG (46 μV/e−) yielding 0.76 e− r.m.s. average read noise [46]. The CMS technique was also explored with low temperature (because of dark current considerations) and achieved limited photoelectron counting capability [44,47,51]. However, the CMS technique with a large number of samples is not feasible for QIS application due to its relatively low speed.

Our approach to achieve deep sub-electron read noise is to improve CG and reduce SF transistor noise. Since our first report of success with this approach [14,17] other groups have also reported success at achieving deep sub-electron read noise (and photon counting) without the use of avalanche gain [50,51]. Improvement of CG was also reported in [62] leading to 0.46 e− r.m.s. read noise, just short of what is needed to demonstrate photoelectron counting.

The photoelectron signal is converted to a voltage signal for readout using the capacitance of the floating diffusion (FD) node. The voltage signal generated by one photoelectron is given by:

(15) $$CG=\frac{q}{C_{FD}}$$

where q is the elementary charge of one electron and $C_{FD}$ is the node capacitance of FD that includes several major components: depletion capacitance between FD and substrate, overlap capacitance between FD and the transfer gate (TG), overlap capacitance between FD and reset gate (RG), SF effective gate capacitance, and inter-metal capacitance. To improve CG, the capacitance of FD needs to be reduced. Note that the reduction of FD capacitance may lead to reduction of FWC in conventional CIS, but it is not a concern for a jot device as the required FWC is very small.

### 5.2. High CG Pump-Gate Jot Devices

**Figure 9:**
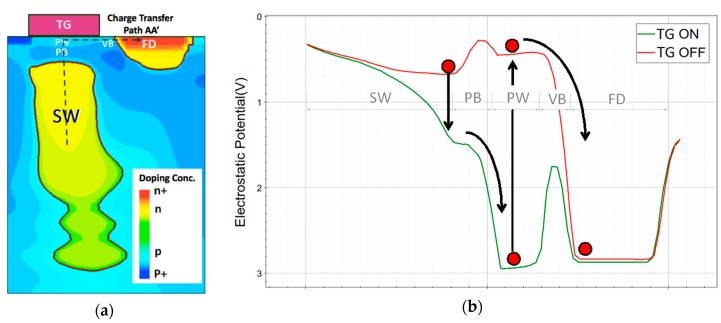
(**a**) PG jot cross-section doping profile from TCAD simulation; (**b**) Electrostatic potential curve along charge transfer path AA’. Both are presented in [13].

Depending on the process feature size and layout design, the overlap capacitance between FD and TG in a CIS pixel can be 0.3 fF or higher, especially in a pixel with a shared readout structure. A pump-gate (PG) technique was developed by our group to eliminate the overlap capacitance between FD and TG without affecting complete charge transfer [13]. A cross-section doping profile of a PG jot device is shown in Figure 9a. A distal FD is formed with no spatial overlap with TG. With different doping concentration in PW, PB and VB regions, two built-in electrostatic potential steps are formed, as shown in Figure 9b. The photoelectrons accumulate in SW during the integration period. During this period, dark current generated directly under TG at the Si-SiO_2_ interface, is blocked from flowing to SW by a barrier, and instead dark current flows to FD. As a result of SW being an n-region fully surrounded in 3D by single crystal p-type silicon, dark current is extremely low. For readout, FD is reset and sampled, and then integrated carriers in SW are transferred to the PW region under TG as TG is turned “on” by a positive bias, and then transferred to FD in a “pump” action when TG is turned “off”, since a built-in barrier prevents their return to SW. With the transferred charge, FD is sampled a second time for correlated double sampling (CDS). The PG jot device has a FWC of about 200 e− and can achieve lag-less charge transfer [18].

**Figure 10:**
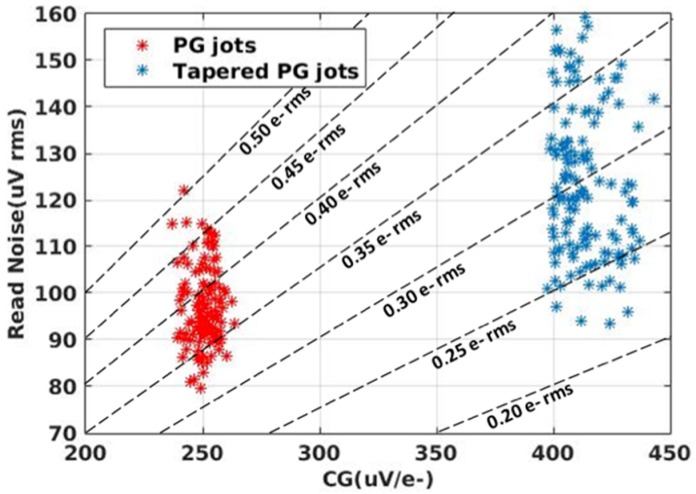
Scatter plot of voltage read noise vs. CG for PG jots and TPG jots. The read noise in e− r.m.s. levels are shown with dashed lines. Presented in [18].

A tapered RG technique was developed to reduce the overlap capacitance between FD and RG, which uses STI to shrink the width of reset transistor on the FD end. The use of the tapered RG (aka tapered PG (TPG) jot) significantly increased conversion gain from 250 μV/e− to over 400 μV/e− and helped reduce read noise from approximately 0.33–0.45 e− r.m.s. range to the 0.22–0.35 e− r.m.s. range as shown in Figure 10. The variation in read noise may be due to fluctuations in the energy levels of traps in the readout transistor, the total number of traps, and other random factors.

**Figure 11:**
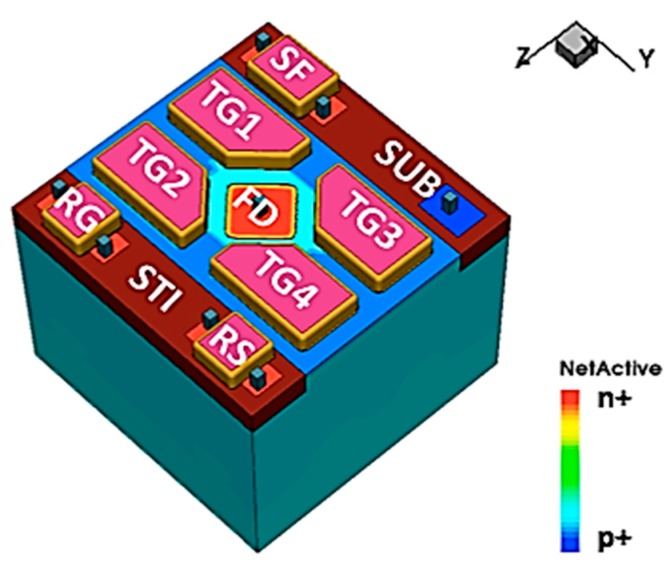
TCAD 3D model of a 4-way shared readout jot, from [20].

The pump-gate technique enables the implementation of shared readout structure (shared PG jot) without adding overlap capacitance due to the distal FD, and the 3D TCAD model of a 4-way shared readout PG jot is depicted in Figure 11. The shared readout jot has a more compact layout design with 1 μm pitch, but since FD needs to be connected to the SF, more inter-metal parasitic capacitance is added to FD, which yields a mildly lower CG.

Both PG jot and TPG jot (PG jot with tapered RG) were designed and fabricated in the TSMC BSI 65 nm process. The fabrication followed baseline process with implantation modifications, and no extra mask was required. The TPG jot pitch is 1.4 μm and has 410 μV/e− CG (0.39 fF FD capacitance), the non-shared PG jot pitch is 1.4 μm and has 250 μV/e− CG (0.64fF FD capacitance), and the 4-way shared PG jot pitch is 1 μm and has 230 μV/e− CG (0.7 fF FD capacitance). As expected, extremely low SW dark current (0.1 e−/s at RT) was measured and almost lag-less (<0.1 e−) charge transfer was achieved. The measured characteristics of jot devices are listed in Table 1.

### 5.3. Photoelectron Counting Capability

**Figure 12:**
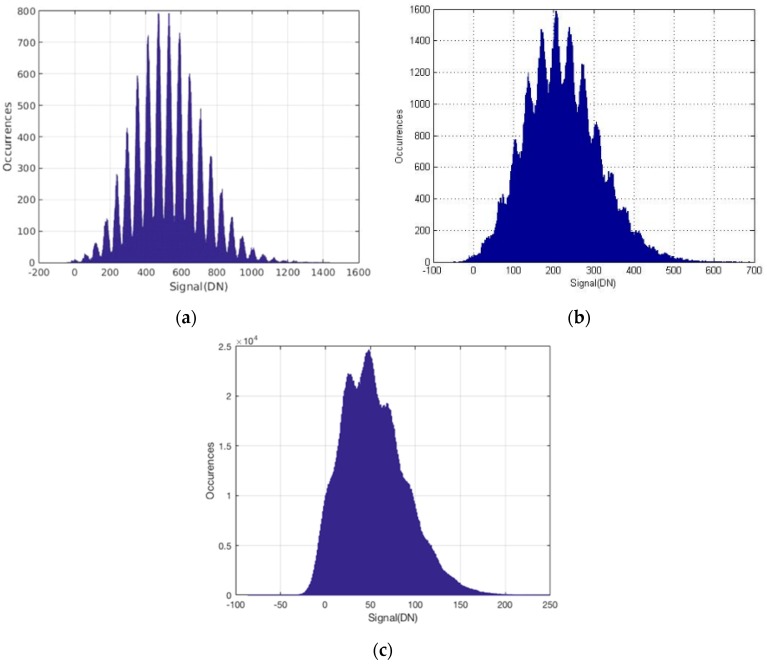
(**a**) PCH of a “golden” TPG jot with 0.22 e− r.m.s. read noise for a quanta exposure of 9. Presented in [18]; (**b**) PCH of a PG jot with 0.32 e− r.m.s. for a quanta exposure of 6.5. Presented in [17]; (**c**) PCH of a shared readout PG jot with 0.42 e− r.m.s. read noise for a quanta exposure of 2.4. Presented in [20].

Both the PG and TPG jots are demonstrated to have deep sub-electron read noise, and the PCH-VPM method was used to characterize their photoelectron counting capability [17,18]. The jots in each 32 × 32 array were readout by single CDS under room temperature (RT). TPG jots have an average read noise of 0.29 e− r.m.s., or 129 μV r.m.s. voltage noise, and a “golden” TPG jot achieved 0.22 e− r.m.s. read noise. The PCH of the “golden” TPG jot is depicted in Figure 12a. It was the first time that a CIS pixel without avalanche gain achieved deep sub-electron read noise and photoelectron counting capability. PG jots have an average read noise of 0.38 e− r.m.s., or 95.3 μV r.m.s. voltage noise. Shared readout PG jots have an average read noise of 0.48 e− r.m.s., or 110 μV r.m.s. voltage noise [20]. The PCHs of these jots are also shown in Figure 12b,c.

**Figure 13:**
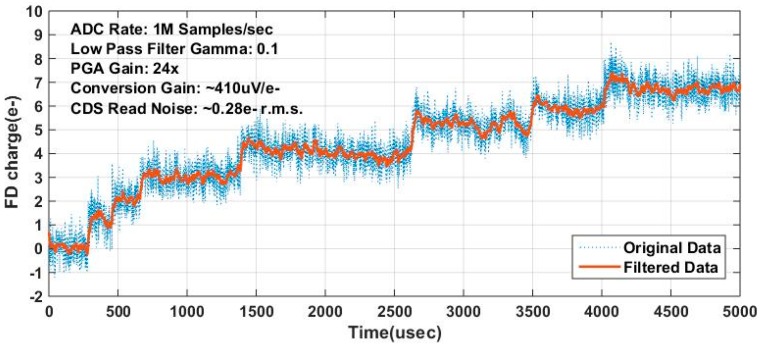
Illustration of photoelectron counting. The signal is the continuously sampled FD voltage from a TPG jot (with 0.28 e− r.m.s. read noise when operated in a CDS mode.) The FD voltage was changed by photoelectrons from SW (and possibly dark generated electrons.) Each single electron generates a fixed voltage jump on FD, and with deep sub-electron read noise, the electron quantization effect is visible.

A more straightforward method was also used to illustrate the photoelectron counting capability of TPG jot. The TPG jot was kept in an integration state under a low illumination and the FD voltage was read out continuously. The quantized voltage steps generated by photoelectrons (and possibly by some thermally generated electrons) can be clearly seen in Figure 13, in which the FD voltage (*y*-axis) is normalized by CG. This is a very basic electrical engineering demonstration of putting one electron on a capacitor and seeing a step in the voltage, but we have not found many prior examples of such an elementary measurement in the literature. It is possible the unfiltered noise in Figure 13 is related to RTS but detailed exploration of this noise has not yet been performed.

### 5.4. Jot Device with JFET SF

It was noticed that although the TPG jot yielded a lower read noise than the PG jot, it actually had higher voltage noise. This effect is believed to be caused by a smaller SF gate area in the TPG jot. As the SF gate capacitance can dominate the total FD capacitance in the PG jot, smaller SF area can provide a higher CG, but also makes the SF more susceptible to the random fluctuation caused by interface traps and leads to increased 1/*f* noise and RTS. With this tradeoff between gate capacitance (that is, CG) and SF voltage noise, further reduction of read noise becomes challenging. The scatter plot in Figure 10 suggests that further reduction of SF size will not allow us to achieve 0.15 e− r.m.s. read noise even with CG of 1 mV/e−. Generally, to achieve the ultimate goal of high accuracy photoelectron counting, more innovation is needed for the jot device to reduce noise or increase conversion gain.

A one-transistor single-electron field effect transistor (SEFET) was proposed as a possibly jot device by earlier work at Samsung [5]. This device used direct collection of photoelectrons in the gate of a junction field effect transistor (JFET) to modulate the current flow of the transistor with CDS performed by resetting the gate back to a fully depleted state. The goals were both small jot size and use of a JFET with high CG and low channel noise to implement low read noise. Only preliminary simulations were performed on the device before the QIS work was abandoned at Samsung.

**Figure 14:**
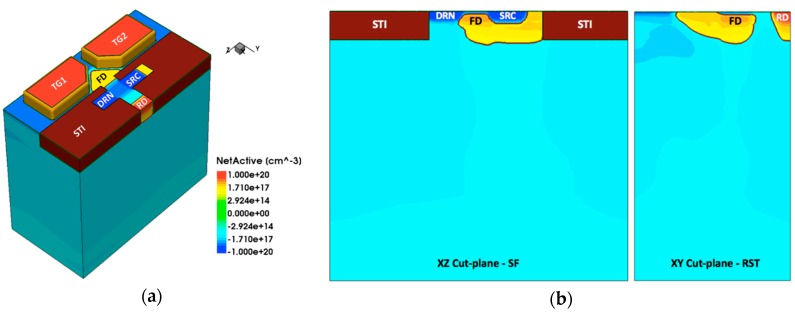
(**a**) 3D doping profile of JFET jot device from TCAD simulation; (**b**) Cross-section doping profiles of JFET SF region.

A jot device with an in-jot JFET SF has been explored with TCAD to address the dilemma in PG jots [23,26]. The doping profile of this device is shown in Figure 14. In this device, FD is the n-type doping well located underneath a p-type shallow channel in the JFET SF, and it also functions as the gate of SF. As photoelectrons are transferred from SW to FD, the potential change in FD modulates the depletion region width in the channel, so as to affect the effective channel depth. With the JFET working as a SF, the source (SRC) is biased by a current source and the drain (DRN) connected to ground. Working in saturation mode, the source voltage would follow the gate (FD) voltage. In the PG jot device with a MOSFET SF, FD is connected to the gate of SF through metal wire, and in order to form an Ohmic contact, FD needs to be heavily doped. In this device, since FD is merged with the gate of SF, no metal connection is needed, so the doping concentration of FD can be much lower, which helps reduce the depletion capacitance between FD and substrate. Also, in a MOSFET SF the gate capacitance is relatively large as a result of the extremely thin gate oxide, but in the JFET SF it is replaced by a much smaller junction capacitance between gate and channel. To further reduce the FD node capacitance, a punch-through reset diode is used in this device. Under this mechanism, FD would be reset when a positive pulse is applied on reset drain (RD). Comparing to CIS pixels with punch-through reset [51,63] taking advantage of the small FWC needed for QIS application, the reset state RD voltage can be much lower (e.g., 2.5 V), and FD would be reset to about 1 V to provide enough FWC. As a result of the reduction in FD capacitance, the JFET jot yields a CG of 1400 µV/e− according to TCAD simulation. Similar to a conventional JFET, this device gate does not interact with channel on the surface interface, which could lead to reduced 1/*f* noise and RTS. Other JFET-based readout devices are also under investigation. Generally, the features of high CG and potentially low noise makes this device a promising candidate to achieve the desired 0.15 e− r.m.s. or less read noise.

### 5.5. Color and Polarization Filters

For many applications, color filter arrays (CFAs) are needed to enable color imaging. In this case, the bit planes can be separated by color into groups and processed independently, and then re-fused for a full color image. Generally, cubicle sizes for the color groups need not be the same and may facilitate particular improvements in image quality. Color processing could also be performed for each combined bit plane followed by cubicle processing. The options are certainly broad but mostly unexplored.

**Figure 15:**
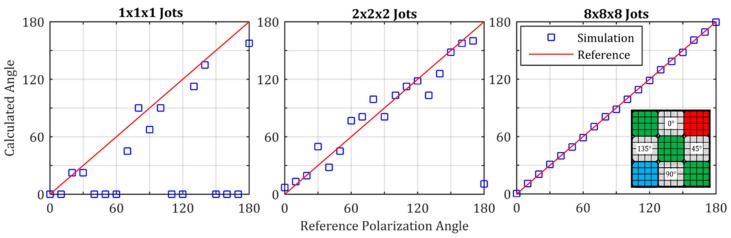
Illustration of polarization angle extraction from various sized cubicles of jots under each polarization-angle filter, from one Monte-Carlo simulation iteration (*H* = 1) for each reference angle. Inset shows 4 × 4 jots under both polarization-angle filters and color filters, to create a 3 × 3 super-kernel to extract polarization and color information from a single-bit QIS. From [24].

For SDL jots, one can consider microlenses and color filters that cover multiple jots (e.g., 2 × 2) since diffraction will likely result in optical resolution lower than the jot pitch [1,64]. Color crosstalk was analyzed in [20] for example, and a new color filter array pattern to ameliorate the impact of color crosstalk was proposed and analyzed in [12]. Polarization filter gratings can also be applied to jots, or groups of jots to select particular polarization of photons [24]. For example, 4 polarization filters formed by gratings, corresponding to 0°, 45°, 90°, and 135° polarization selection angles can each be placed over a group of jots, e.g., 4 × 4 jots under each filter. Color filters can also be adjacent to the polarization filters to form a 3 × 3 super-kernel of filters for polarization and color as shown in the inset to Figure 15. Thus, both color and polarization information can be obtained from the 12 × 12 × *t* super-cubicle of jots, with accuracy dependent on exposure and cubicle size.

## 6. Low-Power and High-Speed Readout Circuits

The principal challenge addressed in this section is the design of internal high-speed and low-power addressing and readout circuitry for the QIS. A QIS may contain over a billion jots, each producing just 1 mV/e− of signal, with a field readout rate 10–100 times faster than conventional CMOS image sensors.

### 6.1. Readout Circuits for Single-Bit QIS

To implement the single-bit QIS ADC, the inherent random offset in a comparator and latch circuit must be overcome to permit practical use of a 500 μV comparator threshold voltage. This traditionally requires additional gain and concomitant power dissipation. For low power, a charge-transfer amplifier (CTA) approach was taken [10]. Minimizing the power dissipation was achieved by using a 4-stage charge-transfer amplifier (CTA) as a gain stage in the analog readout signal chain. Use of CTA technique implemented in pathfinder test chips have resulted in a significant improvement in an energy-per-bit figure of merit (FOM) compared to previous work, although detailed comparison is complicated.

**Figure 16:**
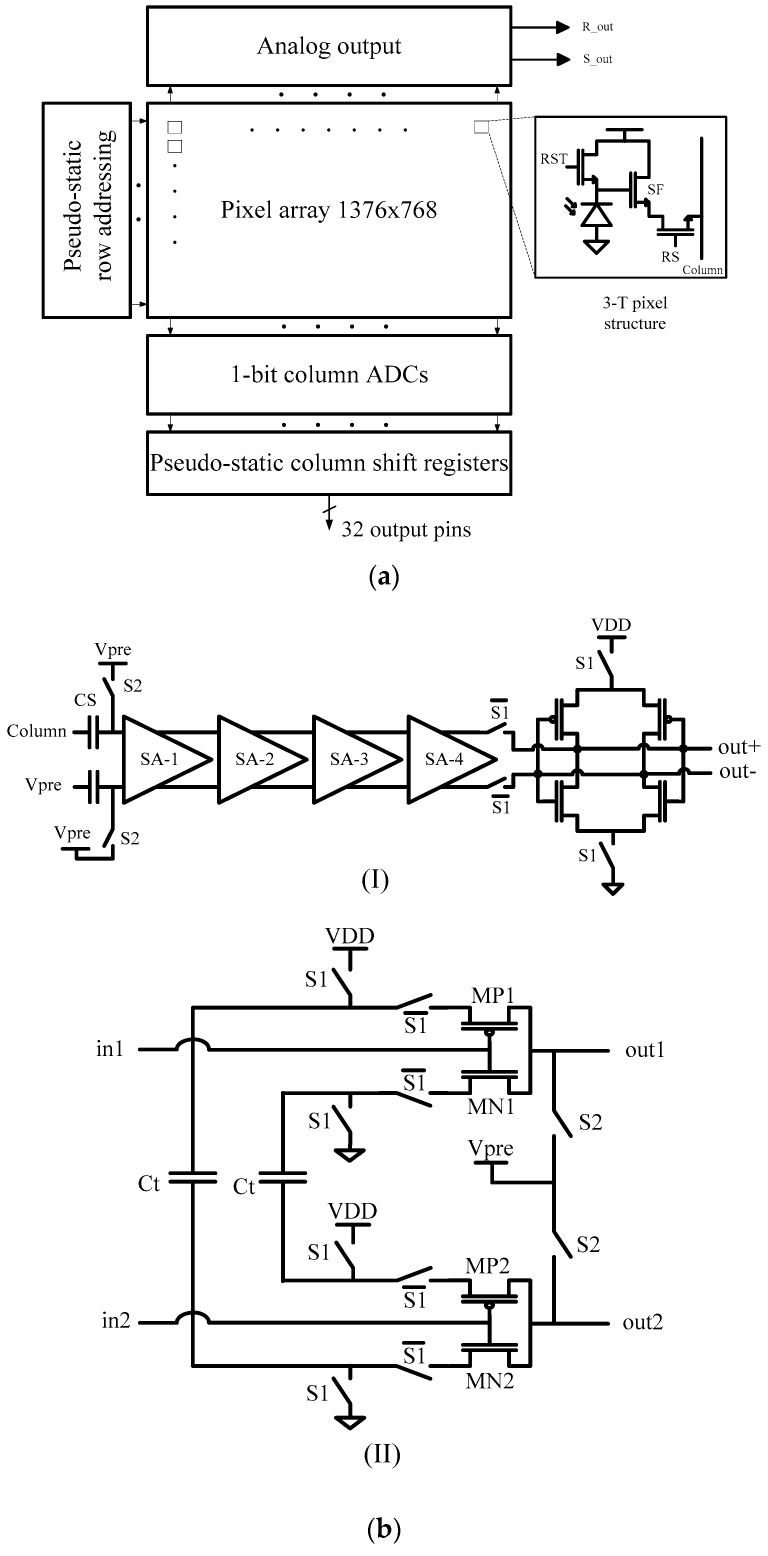
(**a**) Architecture of the 1Mpixel pathfinder image sensor; (**b**) (I) 1-b ADC based on a cascade of sense amplifiers and a single D-latch comparator; (II) Schematic of each sense amplifier that is implemented as a differential charge transfer amplifier.

In the first test chip, low-power readout circuits based on the CTA were implemented in a 1000 fps megapixel binary imager [15,19]. The architecture of the 1 Mpixel pathfinder image sensor is shown in Figure 16a. The 1376 (H) × 768 (V) pixel image sensor uses a partially-pinned photodiode, 3.6 µm 3T pixel, and readout architecture implemented in the X-FAB 0.18 µm process. The sensor is operated in a single-row rolling-shutter mode so true correlated double sampling (CDS) can be utilized. This means that when a particular row is accessed, it is first reset, allowed to briefly integrate a signal, and then read out before moving to the next row. However, to achieve 1000 fps, this leads to extremely short integration times (i.e., <1 µs), useful only in the lab. To characterize the pixels, lower frame rates were used.

A column-parallel single-bit ADC using a CTA-based design detects a minimum 0.5 mV output swing from the pixel (Figure 16b). The ADC is capable of sampling at speeds of 768 kSa/s. The sensor operates at 1000 fps, which corresponds to a row time of 1.3 µs, a signal integration time, T_int_, of 0.9 µs, and an output data rate of 1 Gb/s.

The final specifications of the image sensor are shown in Table 2. The power consumption of the entire chip (including I/O pads) is 20 mW. Total power consumption of the ADCs is 2.6 mW which corresponds to 1.9 µW per column. The row addressing circuits including the buffers consume 0.73 µW per row, whereas the column shift registers dissipate 2.3 µW per column. The ADCs working in tandem with digital circuits consume an average power of 6.4 mW. It is also noted that in the QIS, input offset at 3σ must be less than 1/2 VLSB (=0.5 mV for this chip) which requires additional power dissipation. The FOM of the pathfinder chip is 2.5 pJ/b.

The second test sensor explores the low-power readout circuits needed for a 1040 fps gigapixel binary image sensor [23,25]. Due to limited available area on the die, only 32 of the columns (12,000 pixels in each column) and 16 1b-ADCs were implemented in this test chip. Since the column parallel architecture is used, the power consumption of a column can be multiplied by 2 × 42,000 to estimate the expected total power consumption of a gigajot QIS. This imager was implemented in a 65 nm BSI CIS process. Pixel pitch is 1.4 µm pitch, and 4-way-shared PPD pixels are used in the imager. The same structures of the sense-amplifier and 1b-ADC (size of the transistors and capacitors are scaled down) are implemented in this test chip.

The average power consumption per column (biasing a column with 24,000 pixels and a sense-amplifier and a 1b-ADC) is 68 μW. It is estimated that the power consumption of a gigapixel QIS imager, (ADCs and column biasing) would be approximately 2.85 W. The FOM of the sense-amplifiers and ADC is 0.4 pJ/b. Comparing the power consumption of the ADC that is used in the first single-bit chip (FOM = 2.5 pJ/b) with the ADC in this work, shows that using more advanced technology node (65 nm in this work and 0.18 µm in the first test chip) yields 6× improvement in FOM.

### 6.2. Readout Circuits for Multi-Bit QIS

Conceptually, once input-referred read noise is low enough to count a single photoelectron reliably, counting multiple photoelectrons with the same photodetector and readout structure and a low-bit-depth ADC also becomes practical, allowing implementation of a multi-bit QIS. The ADC digital value is the number of photoelectrons in the jot.

Increasing the bit depth of a jot from single-bit to *n* bits allows the field readout rate to be reduced while maintaining constant flux capacity. Thus, while ADC energy per readout is increased by increasing the jot bit depth, the power dissipation increase is mitigated or negated by the reduced field readout rate. The multi-bit QIS approach also addresses the column limited bandwidth issue, where in single-bit QIS imager, since the integration time is shorter than the integration time in multi-bit QIS, imaging throughput is limited. We have explored several variations of multi-bit QIS architectures, including single-slope, cyclic, and successive approximation ADCs implemented in 180nm CIS process [14]. Results are promising and will be reported in a future publication.

### 6.3. Stacked QIS

A stacked QIS addresses the limited bandwidth problem of the source-follower amplifiers in the pixels or jots. In the stacked QIS approach, more than one substrate or layer could be used to implement the readout circuits. These layers are stacked over each other with bonding interconnections. To readout the jots, the readout and image processing circuits are implemented on the separate substrates.

**Figure 17:**
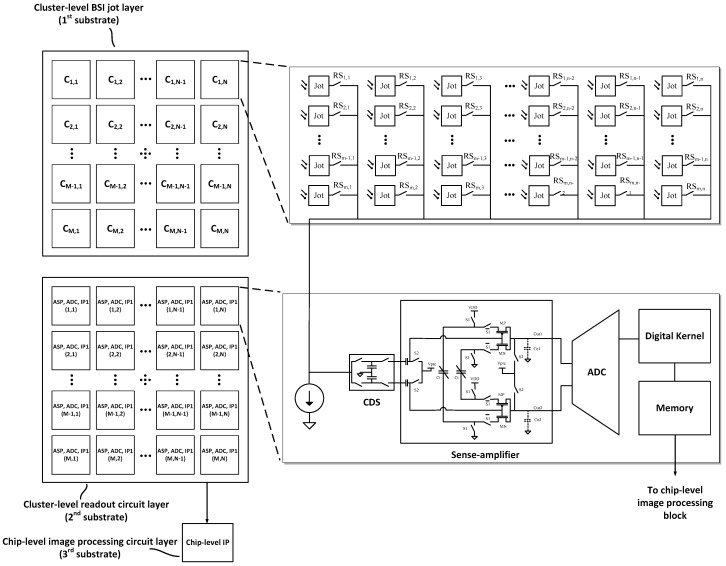
Block diagram of jot clusters, readout circuits and image processing layers.

A stacked QIS may consist of a billion jots which are organized as an array of M row and N column jots [23]. A cluster of jots is defined as a sub-array of m rows and n columns of jots. Figure 17 shows one example of a simplified schematic of a cluster of jots, their analog readout circuits and chip-level signal or image processing units. In each cluster, the RS switches turn on and off sequentially and only one RS switch is connected to the column bus in a cluster at a time. During the selection of one jot, the reset and signal voltage levels are stored on the correlated double sampling (CDS) unit. A differential CTA amplifies the signals stored in the CDS on the level which is bigger than input referred offset and input referred noise of the ADC. All the clusters function in parallel. ADC can be single-bit or multi-bit, based on the readout structure of the entire image sensor system. After quantization of the signal by the ADC, simple digital processing is done on the digital signal by image processor (IP1) and the output is saved in a memory. The simple digital process can be an adder or a digital convolver. The next ADC output, which is the quantized output of the subsequent jot, is summed or convolved with the value stored in the memory. This process continues until all the jots in the cluster have been readout. At this moment, the value stored in the memory, and all other clusters memories are transferred to a chip-level image processor for further processing. After reading one cluster of jots, the clusters readout is re-done for the next frame. By using this method, the bandwidth of the columns in clusters are wide enough to produce thousands of frames per second while consuming very low-power.

As an example, in a gigajot, 1000 fps QIS with 16:9 aspect ratio, with cluster size of 32 (m) × 32 (n), there are 42,000 columns (N) and 24,000 (M) rows of jots and 984,750 clusters as 750 row and 1313 column.

In this system there are 984,750 current sources, CDSs, SAs, ADCs, IP1s, 256-bit memories and one chip-level image processor. The sampling rate of the CDS, SA, ADC, IP1 and memory is 1 MSa/s. Considering 2 W as the power budget for entire chip, 0.5 W may be consumed in chip-level image processing and pad frame, and the rest of 1.5 W budget provides almost 1.5 μW per cluster. Using a more advanced CMOS process such as a 45 nm technology node, charge transfer circuits in the analog domain and sub-threshold regime operation in the digital domain, we estimate it is possible to design the blocks for each cluster to consume less than 1.5 μW power.

It should be mentioned that by using a digital kernel and memory, the output data rate can be significantly reduced, although post-readout processing flexibility is reduced. In the above example, if no image processing was implemented on-chip then the output data rate is about 1 Tb/s; whereas by using simple digital kernels in each cluster, the output data rate could be reduced, for example, to about 8 Gb/s. Using a 3rd stacking layer for chip-level image processing could reduce the output data rate to similar data rates as in conventional cameras.

## 7. Conclusions

This paper has presented a review of progress to date on Quanta Image Sensor made by the group at Dartmouth and others, as well as a brief review of related activity. Much progress has been made since 2012 when work started in earnest at Dartmouth. Implementation of all the critical elements of the QIS has been demonstrated, including image formation, photon-counting jots, and low-power readout electronics. Demonstration of megajot QIS arrays is possible over the next year or two.

## Figures and Tables

**Table 1:** Summary of characterization results of PG jot devices.

Quantity	TPG Jot	Non-Shared PG Jot	Shared PG Jot
CG	410 μV/e−	250 μV/e−	230 μV/e−
Read Noise	0.29 e− r.m.s. (129 μV r.m.s.)	0.38 e− r.m.s. (95.3 μV r.m.s.)	0.48 e− r.m.s. (110 μV r.m.s.)
SF Size	0.2 × 0.2 μm^2^	0.2 × 0.4 μm^2^	0.2 × 0.4 μm^2^
Dark Current @ RT	0.09 e−/s (0.73 pA/cm^2^)	0.12 e−/s (0.98 pA/cm^2^)	Not measured
Dark Current @ 60 °C	1.29 e−/s (10.5 pA/cm^2^)	1.26 e−/s (10.2 pA/cm^2^)	0.71 e−/s (11.4 pA/cm^2^)
Lag @ RT	<0.1 e−	<0.1 e−	<0.12 e−

**Table 2:** Specifications of the 1 Mpixel binary image sensor.

Process		X-FAB, 0.18 µm, 6M1P (Non-Standard Implants)
VDD		1.3 V (Analog and Digital), 1.8 V (Array), 3 V (I/O pads)
Pixel type		3T-APS
Pixel pitch		3.6 µm
Photo-detector		Partially pinned photodiode
Conversion gain		119 µV/e−
Array		1376 (H) × 768 (V)
Column noise		2 e−
Field rate		1000 fps
ADC sampling rate		768 KSa/s
ADC resolution		1 bit (VLSB = 1 mV)
Output data rate		32 (output pins) × 33 Mb/s = 1 Gb/s
Package		PGA with 256 pins
Power	Pixel array	8.6 mW
	ADCs	2.6 mW
	Addressing	3.8 mW
	I/O pads	5 mW
	Total	20 mW

## Abbreviations

The following abbreviations are used in this manuscript:

ADC	analog to digital converter
BSI	backside illumination
CCD	charge-coupled device
CDS	correlated double sampling
CFA	color filter array
CG	conversion gain
CIS	CMOS image sensor
CMOS	complementary metal oxide semiconductor
CMS	correlated multiple sampling
CTA	charge transfer amplifier
DR	dynamic range
DRN	drain
DSERN	deep sub-electron read noise
EMCCD	electron-multiplying CCD
EPFL	École Polytechnique Fédérale de Lausanne
FD	floating diffusion
FOM	figure of merit
FWC	full-well capacity
HDR	high dynamic range
I/O	input-output
JFET	junction field effect transistor
MOSFET	metal oxide semiconductor field effect transistor
MTF	modulation transfer function
PCH	photon-counting histogram
PG	pump gate
PW	p-type well
QE	quantum efficiency
QIS	quanta image sensor
RG	reset gate
r.m.s.	root-mean-square
RT	room temperature
RTS	random telegraph signal
SDL	sub-diffraction limit
SEFET	single-electron field effect transistor
SF	source-follower
SNR	signal to noise ratio
SNR-H	exposure-referred SNR
SPAD	single photon avalanche detector
SRC	source
STI	shallow trench isolation
SW	storage well
TCAD	technology computer-aided design
TDI	time delay integration
TG	transfer gate
TPG	tapered reset-gate pump gate
VPM	valley peak modulation
